# Development of a Monitoring Strategy for Laser-Textured Metallic Surfaces Using a Diffractive Approach

**DOI:** 10.3390/ma13010053

**Published:** 2019-12-20

**Authors:** Sascha Teutoburg-Weiss, Bogdan Voisiat, Marcos Soldera, Andrés Fabián Lasagni

**Affiliations:** 1 Institute of Manufacturing Science and Engineering, Technische Universität Dresden, 01062 Dresden, Germany; bogdan.voisiat@tu-dresden.de (B.V.); marcos.soldera@mailbox.tu-dresden.de (M.S.); andres_fabian.lasagni@tu-dresden.de (A.F.L.); 2 Probien-Conicet, Dto. de Electrotecnia, Universidad Nacional del Comahue, Buenos Aires 1400, Neuquén 8300, Argentina; 3 Department, Fraunhofer Institute of Material and Beam Technology IWS, Winterbergstr. 28, 01277 Dresden, Germany

**Keywords:** indirect surface characterization, diffraction analysis, periodic structures, direct laser interference patterning, homogeneity characterization

## Abstract

The current status of research around the world concurs on the powerful influence of micro- and nano-textured surfaces in terms of surface functionalization. In order to characterize the manufactured topographical morphology with regard to the surface quality or homogeneity, major efforts are still required. In this work, an optical approach for the indirect evaluation of the quality and morphology of surface structures manufactured with Direct Laser Interference Patterning (DLIP) is presented. For testing the designed optical configuration, line-like surface patterns are fabricated at a 1064 nm wavelength on stainless steel with a repetitive distance of 4.9 µm, utilizing a two-beam DLIP configuration. Depending on the pulse to pulse overlap and hatch distance, different single and complex pattern geometries are produced, presenting non-homogenous and homogenous surface patterns. The developed optical system permitted the successfully classification of different pattern geometries, in particular, those showing single-scale morphology (high homogeneity). Additionally, the fabricated structures were measured using confocal microscopy method, and the obtained topographies were correlated with the recorded optical images.

## 1. Introduction

It is well-known today that topographical features of surfaces with sizes in the nanometer and/or in micrometer range have a strong influence on their functionality. The most known and well-described example found in the literature is, for instance, the water repellent characteristics of several plants such as the lotus leaves [1]. Furthermore, the observed super-hydrophobic behavior of this plant was explained by the combination of a topographical pattern of features with different length-scales forming a hierarchical or multiple scale geometry as well as the control of the surface chemistry by three-dimensional epicuticular wax tubules [1,2]. Another famous example is the antibacterial and also hydrophobic abdominal skin of the gecko (*Lucasium steindachneri*), properties that are also the result of a hierarchical structured dermis [3]. 

Currently, an important fraction of the scientific community is investigating how to mimic natural examples with multi-scale or hierarchical surface structures, in order to provide technological materials with similar functions. For example, it was shown that specifically manufactured surfaces are capable of producing functionalities such as decorative elements on metals [4,5,6], antimicrobial properties on stainless steels [7,8], drag reduction on airplane wings [9,10] as well as low friction and wear on engine parts [11]. Especially, grating like textures are of interest in manufacturing diffraction optics, performing inversion of diffraction colors or for surface enhanced Raman scattering sensors [12,13,14,15]. For manufacturing such structures, robust and flexible fabrications methods are needed, providing also high resolution. Laser-based ablation methods in general fulfill these requirements, as they can be utilized directly by locally subtracting (ablating) material at the interaction region between the laser light and the material’s surface. Furthermore, they can be also used on ceramics, metals, polymers, glass, and almost in every material that is capable to absorb the laser light at the utilized laser wavelength. Within these methods, Direct Laser Writing (DLW) has become an important manufacturing technique in the industry but is hardly capable to produce feature sizes down to ~5–15 µm since the focal spot size is generally limited by the wavelength-specific diffraction limit. Utilizing suitable process parameters and laser pulse durations in the picosecond and femtosecond range, laser induced periodic surface structures (LIPSS) can be manufactured with sub-wavelength resolution [16,17,18]. An alternative method, which allows a more flexible fabrication of structures with feature sizes even in the nanometer range is Direct Laser Interference Patterning (DLIP) [19,20,21]. This method is based on the interference patterns that are produced when two or more coherent laser beams are overlapped on the materials surface [22]. In this way, the material can be selectively ablated (or modified) at the so-called interference maxima positions, leading to a structured surface with periodic features. Moreover, the geometry of the patterns can be controlled by changing the number of utilized beams as well as beam polarization among other parameters [19,22,23].

However, significant efforts are still necessary in order to characterize the produced topographies in terms of surface quality or homogeneity. In addition, for in-line quality controls, it is necessary to design methods capable to process the required information rapidly and thus allowing to better control the structuring process. Concerning the available methods for the characterization of surface topography, confocal and interferometric analyses are state of the art and widely common. Furthermore, in ISO 25178 part 6, several instruments and procedures are determined for topographical analysis, including stylus instruments, atomic force microscopes as example for scanning probe microscopy, phase shift microscopy, confocal microscopy, focus variation microscopy, structured light projection, stereoscopic scanning electron microscopy and area integrated scatter techniques [24]. The optical approaches mentioned above have not only the advantage of being contactless and non-destructive methods, but also, due to the nowadays available computing power, are capable of faster data acquisition compared to the stylus instruments and scanning probe microscopes [25]. However, due to the serial processing of multiple images or the scanning directives, these methods are still not fast enough to perform the task of inline monitoring or controlling routines of laser processing techniques. Purtonen et al. collected an overview of laser process monitoring approaches, showing that most of the research is developed for the melt pool evaluation and other thermal process properties of laser cladding, direct energy deposition, welding or selective laser melting [26] but not structuring. For indirect characterization of periodic structures, scatterometry is a very fast and, since it is not affected by the Abbe diffraction limit, a high-resolution technique. It is based on the diffractive behavior of reflected or transmitted light interacting with periodic features, forming intensity distributed patterns. These patterns can be then measured and compared with modeled data. For ex situ evaluation of overlay accuracy in semiconductor industry, an uncertainty of 0.5 nm has been showed with commercial available instruments by Smilde et al. [27]. Furthermore, scatterometry has been efficiently utilized in-line for controlling critical dimensions in lithography cluster [28] and controlling the profile of grating structures in resist trimming [29]. Recently, Kreuzer et al. also proposed the utilization of scatterometry for in-line measurement of Roll-to-Roll imprinted periodic groove widths with a precision of 40 nm [30].

Regarding the characterization of DLIP structured surfaces, recently some studies have been performed in order to determine the pattern homogeneity. For instance, using confocal microscopy, a combination of surface parameters, such as the average structure depth of the repetitive features as well as waviness factors, could be established for determining the structure quality [31]. Moreover, using power spectrum analysis of the topographies also measured by confocal microscopy or white light interferometry, additional parameters could be defined [32]. However, these methods require recording the topographical information utilizing high resolution objectives (e.g., 150X) and cannot be applied directly during the manufacturing process due to the needed measurement time of up to several seconds. In consequence, although some concepts have been already developed for characterizing surface homogeneity and topography of repetitive structures, to the best of our knowledge, there is a lack of optical monitoring approaches capable of rapidly providing information about the surface topography as well as to differentiate between homogenous and non-homogenous surface patterns.

In this work, we present an optical approach to indirectly evaluate the quality and morphology of surface structures manufactured with DLIP by recording their characteristic diffraction patterns in reflection mode when the textured surface is irradiated with a low-power coherent source of light. The recorded image by a CCD camera is processed using self-developed algorithms, in order to determine the most important parameters that can be utilized to determine the homogeneity of the patterns. Finally, the topographical results performed with confocal microscopy are compared to calculated evaluation parameters from the CCD images. As an evaluation tool, the present setup could also be helpful to extract material data and understand the underlying mechanisms by which the material forms periodic textures. For instance, recently it was shown that fracture mechanics data can be obtained by analyzing intermittent periodic patterns on the surface of brittle materials [33].

## 2. Materials and Methods 

Due to the relevance of stainless steels materials in several industrial sectors (e.g., food industry [34,35], pharmacy [36], energy supply (e.g., thermal solar power plants) [37]), we focused on the treatment of electro-polished 1.430 stainless steel with a nominal thickness of 1 mm. The used substrates were cut to 55 mm × 85 mm pieces, with a surface roughness of S_a_ = 0.06 µm (measured according DIN-ISO 25178 norm [38]). Before the laser treatment, all samples were cleaned using isopropanol.

**Figure 1:**
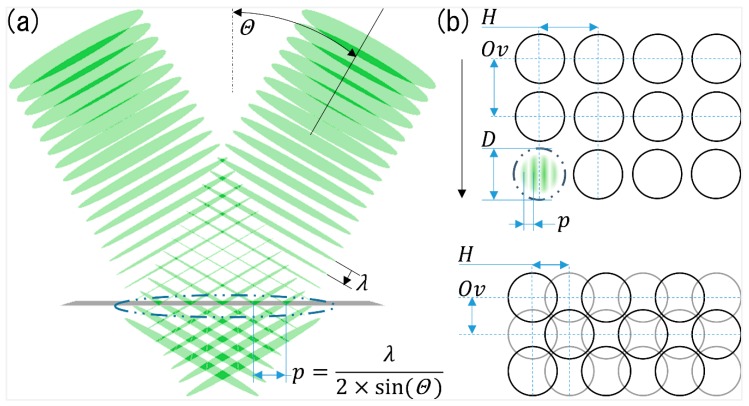
(**a**) Schematic representation of the two-beam DLIP principle for an overlap angle of 2Θ and a laser wavelength λ; (**b**) representation of the strategy used for processing the samples with: D = spot-diameter; p = spatial period; Ov = pulse to pulse overlap; H = hatch overlap. The upper image represents negatives H and Ov overlaps (−25%), while in the lower image, the spots are overlapped by +25% in both directions.

The structuring process was done with a NeoLase Nd:YAG laser (Hannover, Germany), providing a maximal average power of 15 W at a wavelength of 1064 nm. The pulse duration of the system was 70 ps, with a maximal pulse energy of 600 µJ at 1 kHz repetition rate. Using a DLIP optics (Fraunhofer IWS, Dresden, Germany), two individual laser beams were overlapped at the stainless-steel surface (Figure 1a). Using this configuration, a characteristic line-like interference pattern is obtained, whose distance *p* between the interference maxima (or minima) positions is controlled by the laser wavelength λ and the angle between the laser beams 2θ (see Figure 1a), according to Equation (1):

(1) $$p=\frac{\lambda }{2\sin\left(\theta \right)}$$

By automatically varying the interference angle θ between 15.5° and 5.5° for the utilized wavelength, line-like structures with spatial periods between 2.0 to 5.5 µm can be produced, respectively. The used structuring strategy for pulsed laser sources in a DLIP set-up consists on positioning the laser spot with the interference pattern on the sample and separate them a certain distance (vertical and lateral) in order to treat larger areas, which can be exemplarily seen in Figure 1b. Moving the sample with constant speed in one direction by a translation stage, indicated by the black arrow in Figure 1b, and irradiating with a fixed pulse repetition rate results in the treatment of a large area containing the interference structures. Following this direction, the constant distances between each pulse can also be described by the pulse-to-pulse overlap (Ov) which can be calculated as function of the pulse distances and the spot-diameter (D). Accordingly, a value of zero overlap refers to a distance equal to the spot-diameter. The hatch (H) value represents the same percentage relationship but in the direction perpendicular to the primary process direction (Figure 1b). In order to avoid damaging of the periodic structure previously produced, the hatch distance must be always equal to a multiple of the spatial period (p). The geometric layout for negative and positive Overlap and Hatch values (−25% and +25%, respectively) are shown exemplary in Figure 1b.

The surface topography of the structured samples was measured utilizing both non-contact confocal microscopy and white light interferometry methods (Sensofar S Neox 3D Surface Profiler, Barcelona, Spain).

## 3. Results and Discussion

### 3.1. Development of an Optical Measurement System for Periodic Surface Structures

It is well known, that when a periodic structure is irradiated with a coherent light source, a diffraction pattern is obtained. The reflected rays can be classified as diffraction orders, whereby the reflection angle $\Phi_{m}$ is proportional to the order number m, the wavelength λ and the inverse of the spatial period p, as can be seen from the grating equation (Equation (2)):

(2) $$p\sin\phi_{m}=m\lambda$$

for incident irradiation normal to the surface. Therefore, with increasing spatial period, the distance between the diffraction orders decreases when they are projected on a plane. In addition, the orientation in which the diffraction orders are deviated is also aligned to the periodic occurrence of the pattern. Moreover, patterns containing more than one repetitive length scale (as in the case of hierarchical structures) result on multiple diffractions orders based on the different modulations [39]. It is then possible to characterize the surface topography by collecting the diffraction orders with an imaging system. Next, the developed optical system capable of recording the patterns is described. 

**Figure 2:**
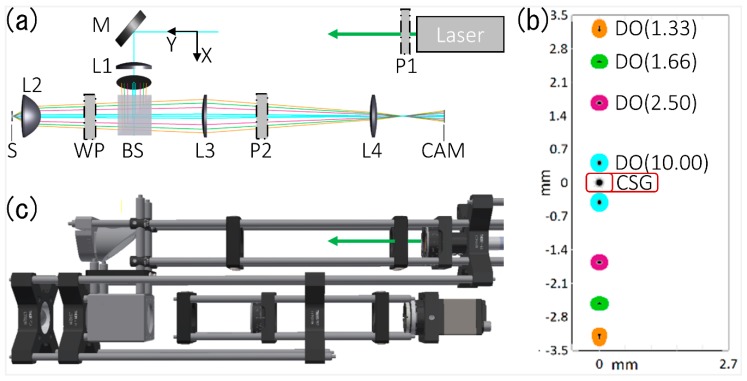
(**a**) Schematic optical set-up of the diffraction measurement system (M, mirror; P, linear polarizer; L, lens; BS, beam splitter; WP, λ/4 wave plate; CAM, Camera) including simulated rays diffracted by gratings with different periods (cyan, pink, green and orange lines); (**b**) Simulated sensor image at the camera (CAM) of the first diffraction orders (DO) for periods of 1.33, 1.66, 2.50 and 10.00 µm; CSG represents the Central Spot Group equivalent to the zero diffraction order; (**c**) CAD model of the fabricated optical system.

The schematic representation of the fabricated system is shown in Figure 2a. As it can be seen, a coherent light source with a wavelength of 532 nm is utilized to illuminate the textured surface, which is indicated in the image as the sample (S). To capture as many diffraction orders (DO) as possible and, in particular, to allow the analysis of patterns with short spatial periods (and thus high diffraction angles), a lens with a high numerical aperture is utilized (L2). For collimating the light reaching the sample surface, the Lens L1 is placed between the laser source and the lens L2. Further, two polarizers (P1 and P2) are used to control the illumination intensity (P1) as well as the reflected intensity (P2) which is guided to the camera (CAM). In this way, the illumination intensity can be adjusted during the experiments. A mirror (M) in combination with a polarized beam splitter (BS) and a quarter-wave plate (WP) are utilized to achieve a separation of the illumination source from the reflected radiation (containing the information about the surface topography) and using an imaging system consisting of two lenses (L3, L4), the reflected light can be collected by the camera.

Using a simulation software (OpticStudio, Zemax LLC, Kirkland, WA, USA) in non-linear mode, all positions for the required optical components were calculated which are listed in Table 1. The simulation results suggest that with the designed measuring setup, the first diffraction orders corresponding to periods as low as 1.33 µm (at an illumination wavelength of 532 nm) can be recorded. As an example, the simulated positions for the first diffraction orders over the CCD camera sensor (CAM) for periods of 1.33 µm, 1.66 µm, 2.50 µm and 10.00 µm are indicated (see Figure 2b). The zero-order (normal to the surface, as the incident radiation) corresponds to the specular reflection and the image area around is defined for the rest of this work as Central Spot Group (CSG). For the mentioned periods in the simulation, the distances of the first-order diffractions to the center of the CCD are 0.5 mm, 1.7 mm, 2.5 mm and 3.1 mm, respectively.

Finally, the measurement system was designed and constructed following the guidelines obtained from the optical simulation and using the components listed in Table 1, as shown in Figure 2c. As it can be seen, the laser source (laser diode CPS532-C2, Thorlabs Inc., Newton, NJ, USA, 532 nm, 0.9 mW) was integrated in the set-up. The polarizers are placed in rotational optic mounts to adjust the illumination and imaging intensity. For further adjustment to real environment, the optic mounts were placed on a cage system with freedom to move separately or in combination of groups.

### 3.2. Direct Laser Interference Patterning of Steel Surface

**Figure 3:**
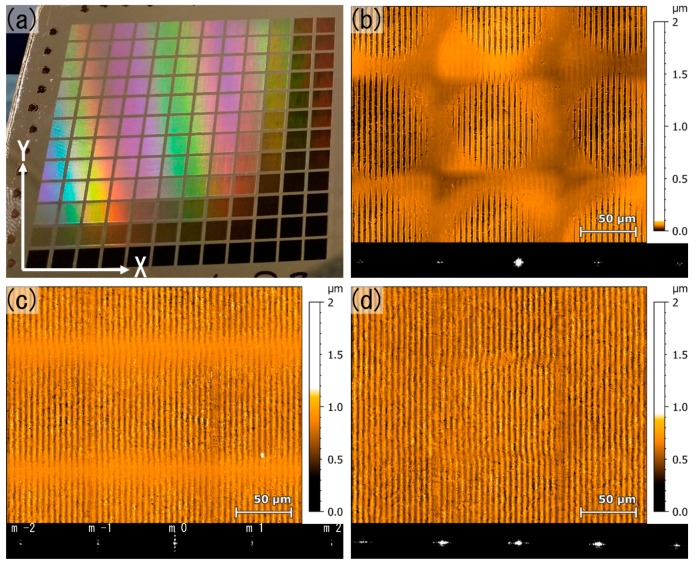
(**a**) DLIP structured stainless-steel sample with a matrix of 12 × 12 fields. The used laser fluence was 1.2 J/cm² and the hatch (horizontal) and pulse to pulse overlap (vertical) were varied in the range from 0% to 99% (with 10% steps till 90%, and then 95% and 99%); (**b–d**) Confocal microscopy images for (**b**) Ov = 0%: H = 0%, (**c**) Ov = 0%: H = 99%, (**d**) Ov = 60%: H = 60%. The insets show the recorded diffraction patterns by the CCD camera with m = −2, m = −1, m = 0, m = 1 and m = 2 (in (**c**)) as indicators for the diffraction orders accountable from the DLIP structures.

Firstly, the electro-polished stainless-steel surface was treated using a constant laser fluence of 1.22 J/cm², varying both hatch-distance (H) and pulse overlap (Ov). For instance, an overlap of 0% means that the separation between the laser pulses was 100 µm, corresponding to the utilized beam diameter (Gaussian shaped). The same criterion was used to describe the hatch-distance in %. The interference spatial period was set to 4.9 µm to decrease uncertainty about possible spherical aberration at small periods. The steel sample was structured in a matrix form with 12 × 12 fields, each with an area of 3 mm by 3 mm, as can be seen in Figure 3a. Overlap (vertical, Y) and Hatch (horizontal, X) was varied from 0% to 99%, respectively (stepwise 0% till 90% in 10% steps and then in addition with 95% and 99%). The shiny colors for some laser processing parameters can be explained by the produced DLIP periodic structures as well as the occurrence of laser induced periodic surface structures (identified in scanning electron microscopy, not shown), which produce a characteristic rainbow color [40,41]. The darker areas in the textured samples correspond to high overlaps, which normally yield deep structures that reduce dramatically the surface reflectance.

Figure 3b–d shows representative images of the surface topography of the laser treated samples, with a fixed spatial period of 4.9 µm, when using different hatch distances and overlaps. For instance, for H = 0% and Ov = 0%, the DLIP spots are clearly separated (Figure 2b), being possible to easily distinguish them. Differently, when the hatch distance is reduced (e.g., H = 99%), keeping the overlap at 0%, a wide area (which is determined by the laser spot diameter of 100 µm) containing the DLIP structures over the whole hatch direction was obtained as shown in Figure 3c. If both, the hatch and overlap distances are decreased, a more homogenous surface pattern is observed, where only the DLIP structures over the whole area are visible, as shown in Figure 3d. In addition to the topography of the produced patterns shown in Figure 3, also the corresponding diffraction images are indicated, which were captured with the CCD camera of the designed optical system. As stated, the distance between the different diffraction orders depends on the distance of the repetitive structures. Thus, the observed diffraction orders (m = −2, m = −1, m = 0, m = 1 and m = 2 as indicated in Figure 3c) correspond to the DLIP structures, while the additional features observed (exemplary the vertical distributed intensity peaks for m = 0 in Figure 3c) correspond to the larger repetitive patterns, which in turn correlate with both the used hatch and overlap distances. Additional information regarding the complex diffraction patterns observed can be found in [42,43].

**Figure 4:**
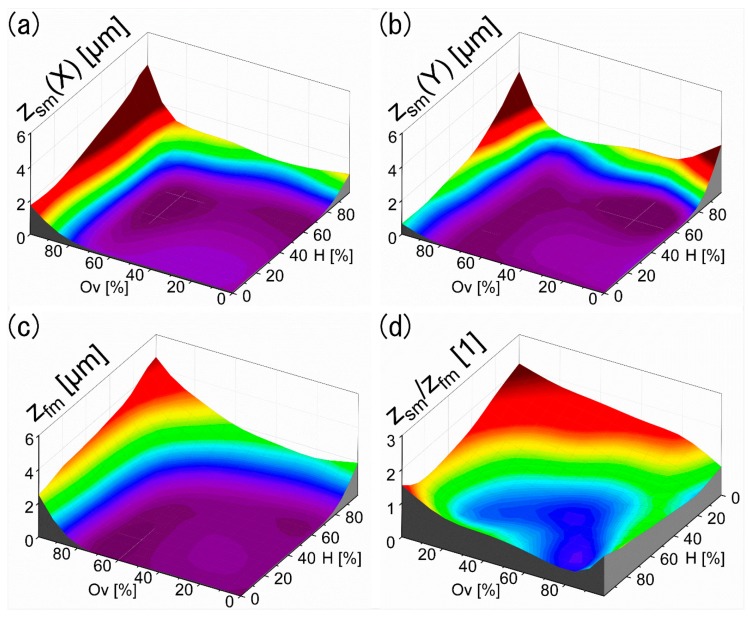
Topographical measurements of structure depths (z) as function of overlap and hatch values. (**a**) Second modulation depth measured along X-orientation Z_sm_(X) and (**b**) Y-orientation Z_sm_(Y); (**c**) mean DLIP structure depth Z_fm_; (**d**) quotient between both depth modulations (Z_sm_/Z_fm_ defined in Equation (3)).

In order to quantitatively characterize the homogeneity of the different periodic structures as function of the used hatch distances and overlaps, both the mean structure depth of the periodic structures, corresponding to the interference pattern (called from now on “first modulation”, Z_fm_) as well as the structure height of the larger surface structure (denoted as “second modulation”, Z_sm_) were determined. These values are plotted for the second modulation in Figure 4a,b and for the first modulation in Figure 4c. As it can be seen from Figure 4c, the height of the DLIP structures increases with higher overlap and hatch. This can be explained by the number of pulses that are irradiating the same area and therefore the contributed sum of pulse energy (exposure dose or accumulated fluence) facilitating the ablation process, which was already demonstrated elsewhere [44]. For instance, an overlap and hatch of 0% to 80% means an increase from 1 to 16 pulses, while overlap and hatch from 80% to 99% results in 25 to 100 pulses. Figure 4c also shows, that in these experiments, mean structure depths from ~100 nm to ~5 µm could be achieved.

The height of the second modulation (Z_sm_) was measured along to either the orientation according to the X (Figure 4a) or Y (Figure 4b) coordinates as indicated in Figure 3a. Taking into account that the interference pattern is created within a Gaussian beam distribution, it can be expected that more pulses (high overlaps) impinging the material surface can produce deeper valleys [45]. Thus, when the hatch percentage is increased above 70% at constant overlap values, deeper valleys oriented unidirectional along the X-axis (see Figure 3c) are produced, resulting in a significant increase of the second modulation depth Z_sm_(Y) measured perpendicular to the valley orientation as shown in Figure 4b. The same effects occur when increasing the overlap percentage at constant hatch distances. Thus, in this case the second modulation depth measured along X Z_sm_(X) (Figure 4a) increases, as the valleys are oriented perpendicular again. In comparison, Z_sm_(X) reaches a minimum of 2 µm at 99% overlap, while Z_sm_(Y) only reaches a minimum of 0.7 µm at 99% Hatch. This can be explained by the hatch distance correction, necessary to avoid destroying the line-like DLIP structures as explained in Section 2 and the resulting total energy input to the surface. An assigned hatch H from 95% and 99% corresponds to an actual hatch distance of 9.8 µm (two times the spatial period of 4.9 µm) and 4.9 µm (equal to spatial period), respectively, whereas for the same overlap Ov percentages of 95% and 99% the corresponding spot-to-spot distance observed on the structured sample is 5 µm and 1 µm, respectively. For overlaps and hatches below 85% (corresponding to less than 45 pulses), the second modulation depth in either direction is below 500 nm. 

Since a homogenous DLIP structure can be characterized by having large depths for the periodic structure (Z_fm_) as well as simultaneously low depths for the large pattern modulation (Z_sm_), the ratio between both parameters (Z_sm_/Z_fm_) was calculated using Equation (3):

(3) $$\frac{Z_{sm}}{Z_{fm}}=\frac{\sqrt{\left(Z_{sm}{\left(X\right)}^{2}+Z_{sm}{\left(Y\right)}^{2}\right)}}{Z_{fm}}$$

This ratio is shown as function of both hatch and overlap percentages in Figure 4d. Note that the lateral scales, i.e., Ov and H, were flipped for a better visualization. Using this criterion, the samples with low Z_sm_/Z_fm_ ratio correspond to textures where the periodic structure produced by the interference pattern is dominant (see for instance Figure 3d). As it can be seen in Figure 4d, laser textured surfaces with overlap and hatch values below 20% are highly inhomogeneous as the second modulation is more dominant compared to the DLIP structure modulation. For overlaps ranging from 40% to 85% as well as hatches between 40% and 85%, the lowest Z_sm_/Z_fm_ ratios were obtained (down to ~0.3). For overlaps and hatches above 85% the structure becomes inhomogeneous due to the high applied energy leading to a significant deterioration of the pattern uniformity as discussed elsewhere [46].

### 3.3. Characterization of the Laser Textured Surfaces Using the Developed Optical Module

After determining the topographical parameters of the different treated samples as function of hatch distance and overlap, the developed monitoring device was evaluated. The first aim was to determine if it is possible to define different parameters that can be correlated with the structure homogeneity, defined in this work as Z_sm_/Z_fm,_

**Figure 5:**
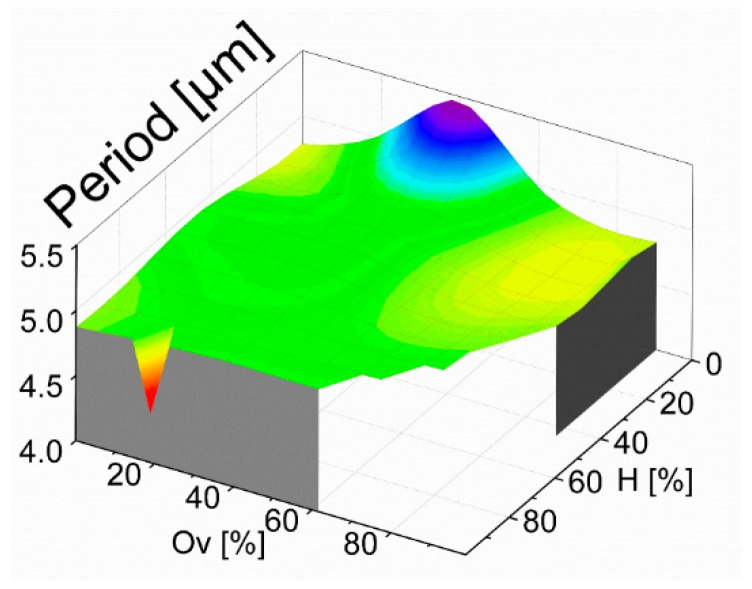
Representation of the spatial period as function of hatch and overlap calculated after calibration of the optical device.

With the whole diffraction pattern recorded for each sample, we first calculated the average pixel distance between the zero order to the minus and plus first diffraction orders corresponding to the first modulation (exemplarily m = −1, m = 0 and m = 1 in Figure 3c), to monitor the periodicity of the manufactured structures. Taking into account all the samples that showed enough intensity irradiating the CCD sensor from the reflected light (a discussion on this regard will be given below), the mean distance was 166.9 pix with a standard deviation of only 4.5 pix. Considering that the obtained standard deviation is only 2.7% of the calculated mean value, it can be stated that the period of the DLIP structure is stable over all the analyzed samples. The calculation was also migrated in the evaluation algorithms utilizing the dilated image of the CCD (visualized in the Supplementary Material S1 and S2), showing the same results. Considering the relationship between the diffraction order angle for a specific diffraction order m (see Equation (2)), as well as by performing a calibration of the system with known periodic patterns, the mean distance between the first order and the zero order of 166.9 pix was found to represent a spatial period of 4.905 µm. Using this information, it was possible to calculate the spatial period of the repetitive pattern as function of the overlap and hatch values as shown in Figure 5. As it can be seen, the detected period oscillated slightly around its mean value and its variation corresponds to a standard deviation of 0.15 µm with two outliers of 4.76 µm (overlap of 20% and hatch of 99%) and 5.78 µm (overlap of 40% and hatch of 0%). For samples where no diffraction image on the sensor could be captured, no periods can be calculated and, therefore, no values are present in Figure 5.

**Figure 6:**
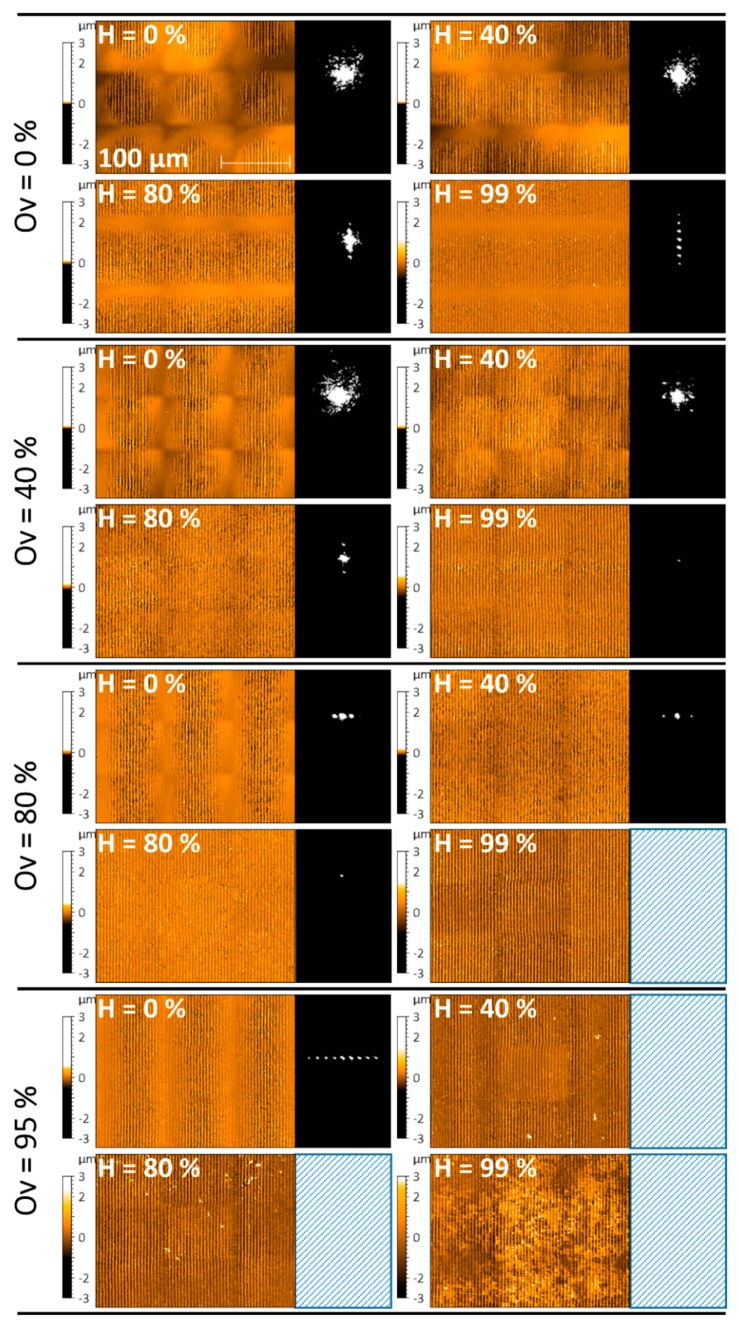
Exemplary selected confocal microscopy images (left) and captured diffraction pattern of the Central Spot Group (CSG) (right) as function of both overlap (Ov) and hatch (H). The blank rectangles indicate that no light intensity could be recorded.

For further evaluation, we decided as a first approach to analyze only the central spot group (CSG). The reason for this is that in preliminary experiments (not shown) the direct reflected zero-order showed the most promising, as in most sensitive and clearly distinguishable images to the naked eye for different modulations. Exemplary images of the CSGs recorded with the CCD camera are shown in Figure 6 for different DLIP processed stainless steel samples, grouped by constant overlap percentages of 0%, 40%, 80% and 95% (Ov-Groups) with hatch parameters of 0%, 40%, 80% and 99%, respectively. 

As it can be seen in Figure 6, for an overlap Ov = 0% (see top images), an increase in the hatch overlap percentage results in an elongated central spot group in the direction parallel to the interference lines, or perpendicular to the large modulation pattern. This can be attributed to the superposition of several laser pulses in the hatch direction. Moreover, it can be clearly seen that for a hatch H of 99%, the CSG shows several spots perpendicular to the hatch direction, since the repetitive large modulation also diffracts the incoming light into well-defined directions. A similar behavior is observed when keeping the hatch overlap H at 0% and increasing the pulse overlap, which can be seen following the upper left images of the Ov-Groups in Figure 6. Since in this situation the second modulation periodicity is dependent on the hatch distance and, therefore, the macrostructure valley orientation is parallel to the DLIP structures, the elongation of the CSG as well as the splitting occurs in the same direction as the diffraction from the DLIP structures. Moreover, the splitting direction occurs in respect to the dominant repetitive orientation of the second modulation; as for overlaps Ov of 40% and hatch H of 80%, the diffraction orders split vertically, but upon inverting the parameters (Ov = 80% and H = 40%), the diffraction orders split horizontally. For certain Ov and H values in the range of 20% to 60%, for instance, Ov = 40% and H = 40%, both splitting directions can be distinguished in a sort of a two-dimensional split diffraction of the CSG. It is worth to mention that on surfaces showing a high homogeneity (such as for Ov and H = 80% or Ov = 40% and H = 99%), the CSG is reduced to a very small spot, since the periodic structure corresponding to the interference patterns acts as a very well-defined diffraction grating.

Finally, in the cases of stainless steel surfaces which were processed at very large overlap and hatch percentages, the developed optical module was not capable of collecting sufficient light intensity since these samples presented a very dark appearance and thus their reflectivity is strongly reduced compared to the other samples (see Figure 3a, on the bottom/right corner).

**Figure 7:**
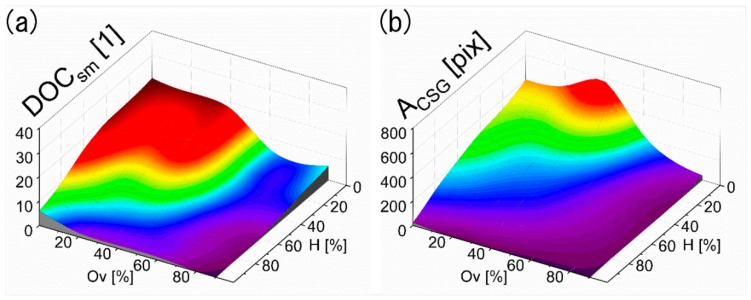
(**a**) Measured diffraction order count of the second modulation (DOC_sm_) and (**b**) total area illuminated of the Central Spot Group (A_CSG_) plotted over overlap and hatch values.

Based on the preliminary information reported in the previous section, for indirectly evaluating the quality and morphology of the produced surface patterns, two figures of merit are defined: (i) the amount of diffraction orders corresponding to the second modulation within the CSG (defined as Diffraction Order Count DOC_sm_) and (ii) the total area of the central spot group (CSG) recorded by the camera sensor (A_CSG_). The developed algorithms used for the calculation of both DOC_sm_ and A_CSG_, are based on OpenCv [47] image processing library and are presented in the supplementary information section. Figure 7 shows (a) the DOC_sm_ and (b) A_CSG_ parameters, as function of the overlap Ov and hatch H. As it can be seen in Figure 7a, the amount of diffraction orders emitted by the second modulation increases with reduced Ov and H values. For example, DOC_sm_ reaches values up to ~20 orders for overlaps and hatches smaller than 40%. Recalling the topographies in Figure 6 with Ov and H below 40%, it is possible to see that significant areas of those samples remain unstructured, and thus, the reflected light (from the illumination system) is mostly directed to the CCD sensor or scattered, leading to a broad central spot surrounded by several smaller spots. In this case, the DOC_sm_ calculation includes the total amount of every separated illumination area detected on the sensor rather than the actual diffraction orders caused by the second modulation. With increasing hatch H and overlap Ov, the structures become deeper (see Figure 4) and the reflected light splits into better defined diffraction orders. For instance, the CSG shows only about 3 individual spots when using H ~ 20% and Ov ~ 60%, as well as for H ~ 95% and Ov ~ 20%. 

As observed in Figure 7b, the area of the CSG A_CSG_ is comparatively large for overlap and hatch values below 40%, due to the same reasons mentioned above for the DOC_sm_ values. As the overlap or hatch values increase, more distinct diffraction spots are recorded causing a reduction of the A_CSG_ parameter. For the most homogeneous structures showing only the DLIP features, the CSG consists only of a single spot with a small area, as explained before. In consequence, small A_CSG_ as well as a DOC_sm_ = 1 are expected. This case was observed in those samples with overlaps Ov and hatch H combinations in the range of 60% to 95% each.

**Figure 8:**
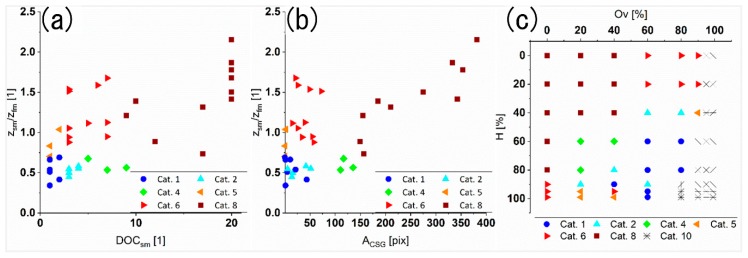
Categorized results of the homogeneity fraction Z_sm_/Z_fm_ (depth of first modulation by the DLIP structure to depth of the second modulation ratio) in comparison to (**a**) Diffraction Order Count of second modulation (DOC_sm_) and (**b**) Area of the Central Spot Group (A_CSG_)); (**c**) Representation of the different category-definitions as function of hatch H and overlap Ov. The different categories are listed in Table 2.

Finally, the measured parameters describing the real surface topography of the different produced patterns from Section 3.1 are compared with the parameters of the diffractive approach. The main objective is to determine the possibility of using the information recorded by the developed optical module for describing the real topography. As it can be seen in Figure 8, the monitoring parameters (DOC_sm_ in Figure 8a and A_CSG_ in Figure 8b) were plotted against the homogeneity coefficient (Z_sm_/Z_fm_ in Figure 4d). For a better understanding of the type of topography representing each experimental data point, the data was categorized as listed in Table 2 and also displayed according to their category with different colors and symbol shapes, depending on the categories defined.

As the goal of the diffractive approach is differentiating between homogenous and inhomogeneous structures, the homogeneity quotient Z_sm_/Z_fm_ boundary was arbitrarily defined as 0.7, resulting in categories #1 to #4 for good homogeneity (below 0.7) and categories #5 to #8 for poor homogeneity (above 0.7). To draw a conclusion, if the developed system can distinguish the homogeneity by the diffraction analysis parameters, each category range is further subdivided in small and large A_CSG_ (separation limit of 100 pix), as well as in few and many DOC_sm_ (separation limit of 2) according to Table 2. Category #10 is introduced for those samples whose diffraction data could not be obtained due to no measurable intensity of reflected light. Category #7 (and also #3) would probably per definition point towards a mirror-like surface with no scattering, which was not recorded by the developed system nor topographically measured at all and, therefore, will not be discussed further. Samples featuring homogeneous structures would be, by the definitions made and relations shown, expected to only show up in category #1 with no splitting due to the second modulation (DOC_sm_ ≤ 2 in Figure 8a) and small illumination area A_CSG_ (<100 pix in Figure 8b). This is in agreement with textured samples previously discussed for overlap and hatch parameters of 60% to 85%, shown in Figure 8c. However, there is a mismatch for samples with slightly varied structuring parameters as seen in Figure 8c (category #5), which suggest a good homogeneity (small illuminated area A_CSG_ and DOC_sm_ of 1 or 2), while actually being inhomogeneous by topographically measurements (Z_sm_/Z_fm_ > 0.7). For these samples, the reflected diffraction pattern has a low intensity which cannot give enough contrast on the CCD sensor, making the additional splitting orders not detectable. This can be also concluded by looking to the illuminated area which for these samples is close to zero (A_CSG_ < 4 pix). Further strengthening this reasoning, the samples classified as #5 type are occurring close to category #6 type samples in terms of their structuring parameters (as seen in Figure 8c). As stated before, a high number of the DOC_sm_ in combination with a small illuminated area can be attributed to splitting of the CSG and therefore to an inhomogeneous structure with a dominant second modulation, which corresponds very well with the samples in category #6 (see Figure 8a).

Category #2 cannot be distinguished from category #6 by the developed system alone, as both would indicate a splitting behavior of the CSG (DOC values > 2) and therefore a significant contribution from the second modulation to the diffraction pattern. For samples of category #6 this is in agreement with the homogeneity factor (Z_sm_/Z_fm_ > 0.7). Although, the topographical data of the samples in category #2 imply a good homogeneity (homogeneity factor ~0.5 in Figure 4d), it can be observed that the developed system clearly detects the second modulation within the diffraction images (exemplary Ov = 40% and H = 80%, as well as Ov = 80% and H = 40% in Figure 6). 

Similarly, category #4 and category #8 would be measured by the optical system as inhomogeneous. In the case of category #8, the reason can be attributed to high scattering and a large illumination area, which corresponds to an only partly structured surface. In turn, for category #4 samples, this can be explained by a relatively shallow surface texture, where even a shallower second modulation (contributing to a low homogeneity factor) has a significant impact on the whole surface morphology and therefore on the diffraction image. 

Possible solutions to enhance the monitoring device detection quality will be evaluated, including a more precise illumination control (to avoid saturation at the CCD camera) combined with a more powerful illumination source, as well as a camera with better dynamic range or fine tuning of the evaluation algorithms. In this work, the structured samples were directly evaluated by topographic criteria alike to other measurement systems like confocal microscopy. The oxidation on the surface or its degree and a possible impact on the measurements have not been considered or studied.

## 4. Conclusions

In this work, an optical approach was developed and constructed for the indirect evaluation of the quality and morphology of surface structures manufactured with DLIP. For testing the designed optical configuration, line-like surface patterns were produced on stainless steel with a periodicity of 4.9 µm utilizing a two-beam DLIP configuration. Depending on the pulse to pulse overlap and hatch distance, it was possible to fabricate complex geometries presenting non-homogenous and homogenous surface patterns.

The developed optical system permitted us to successfully capture reflective features from the textured surfaces that were used for evaluation and monitoring purposes. Different algorithms were developed for processing the captured images, in order to calculate the periodicity of the first modulation, the total area of the central spot as well as the number of diffraction orders that correspond to the second modulation (if any) of the structure. By comparison to topographical measurements, it was shown that the developed approach was capable to distinguish between different structures groups, which were characterized by specific surface parameters. Moreover, in comparison to the topographical measurements and calculations, the system shows more precise detection of existing second modulations for shallow surface structures. However, the measurement quality of the system as for now was found to be limited by the illumination source (low power diode laser) and thus to not permit characterizing patterns with deep structures. Since periodic patterns exhibiting high homogeneity are relevant for many surface functions (for example for surfaces with enhanced optical properties), the presented method will be relevant in the future to monitor the manufacturing process, being capable to ensure high quality as well as to detect process instabilities and new hints to understand the underlying mechanisms by which the resulting textures are produced. These topics will be investigated in the future.

## Figures and Tables

**Table 1:** Parameters and positions (X and Y as indicated Figure 2a) of the different elements utilized for the optical imaging system simulation.

ID	Object	X (mm)	Y (mm)	Parameter
Laser	Coherent light source	0	0	λ = 532 nm, 0.9 mW, 3.5 mm beam diameter
P1	Polarizer	(0)	-	Linear Dichroic, λ = 400–700 nm
M	Coupling Mirror	0	60	For 532 nm wavelength
L1	Lens	20	60	Plano-Convex, N-BK7, f = 75 mm
BS	Beam Splitter	49	60	Polarized for λ = 532 nm
WP	λ/4 Wave plate	(49)	-	Zero order for 532 nm
L2	Lens	49	140	Asphere, N-BK7, f = 16 mm, NA = 0.79
S	Sample	49	146	Textured sample with different spatial periods
L3	Lens	49	−10	Plano-Convex, N-BK7, f = 100 mm
P2	Polarizer	(49)	-	Linear Dichroic, λ = 400–700 nm
L4	Lens	49	−110	Bi-Convex, N-BK7, f = 50 mm
CAM	Camera	49	−160	Camera screen size: 2.7 mm × 3.4 mm

**Table 2:** Definition of categories #1 to #10 for treated samples by the homogeneity factor Z_sm_/Z_fm_, the illumination area of the CSG A_CGS_ and the diffraction order count obtained from the second modulation DOC_sm_. Note that no samples fitted in the categories #3 and #7.

Category #	1	2	3	4	5	6	7	8	10
Z_sm_/Z_fm_	<0.7				>0.7				No Data
A_CSG_	<100		>100		<100		>100		
DOC_sm_	≤2	>2	≤2	>2	≤2	>2	≤2	>2	
CSG example in Figure 6 Ov,H	80, 80	40, 80	-	40, 60 *	40, 99	0, 99	-	40, 0	-

* can be found in supplementary Figure S3.

## Supplementary Materials

The following are available online at https://www.mdpi.com/1996-1944/13/1/53/s1, Figure S1: Evaluation algorithm flow chart and algorithm declarations with examples, Figure S2: Subroutine algorithm of categorization flow chart, Figure S3: Example CSG of category #4.
