# Supplementary figures and images for: Development of a Monitoring Strategy for Laser-Textured Metallic Surfaces Using a Diffractive Approach
Source: Materials (Basel). 2019 Dec 20;13(1):53. doi: 10.3390/ma13010053 (PMC6981385; doi:10.3390/ma13010053)

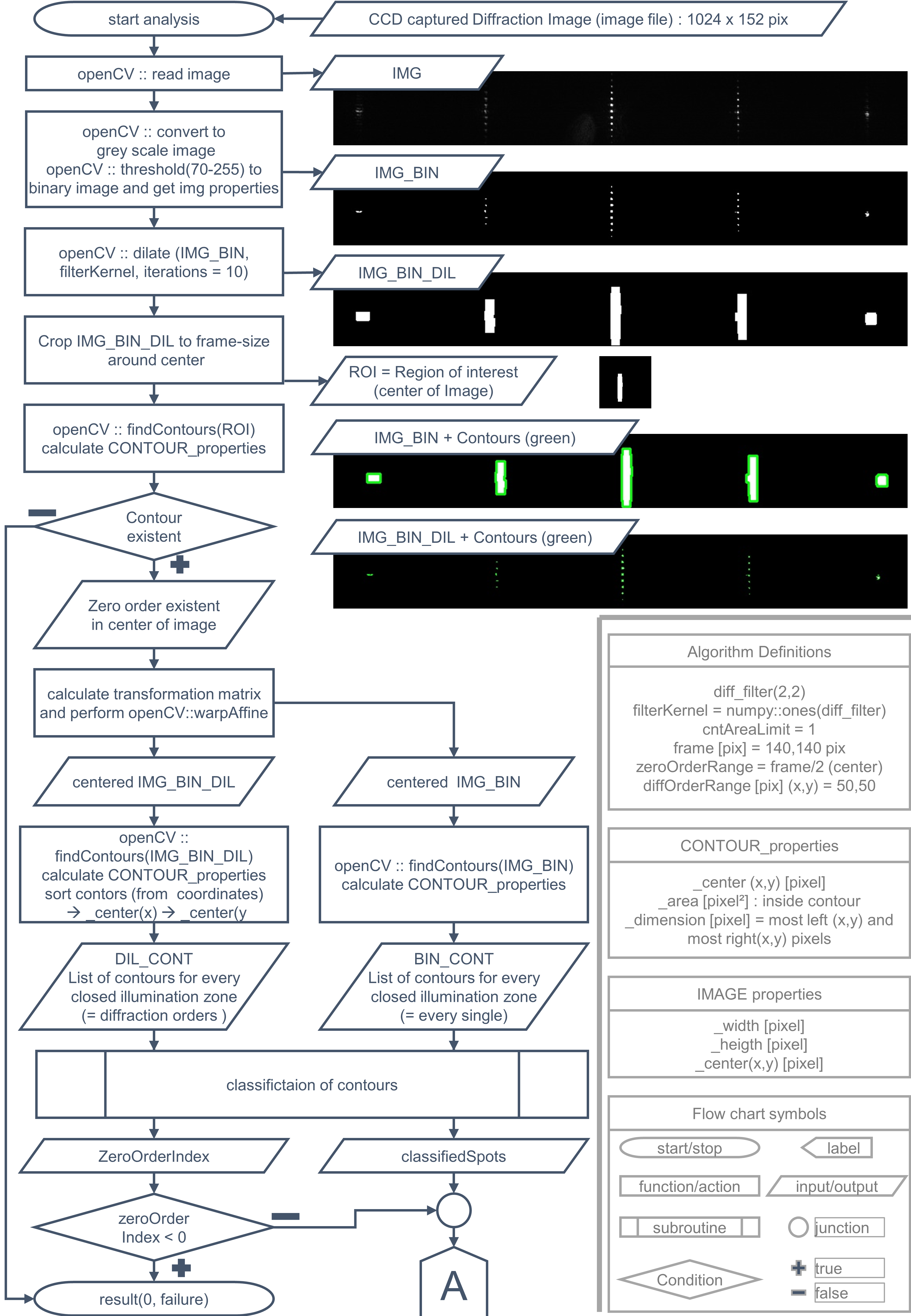

Supplement: Supplementary file 1 [file materials-13-00053-s001.zip › Figure S1 - FlowChart 1_2.TIF]

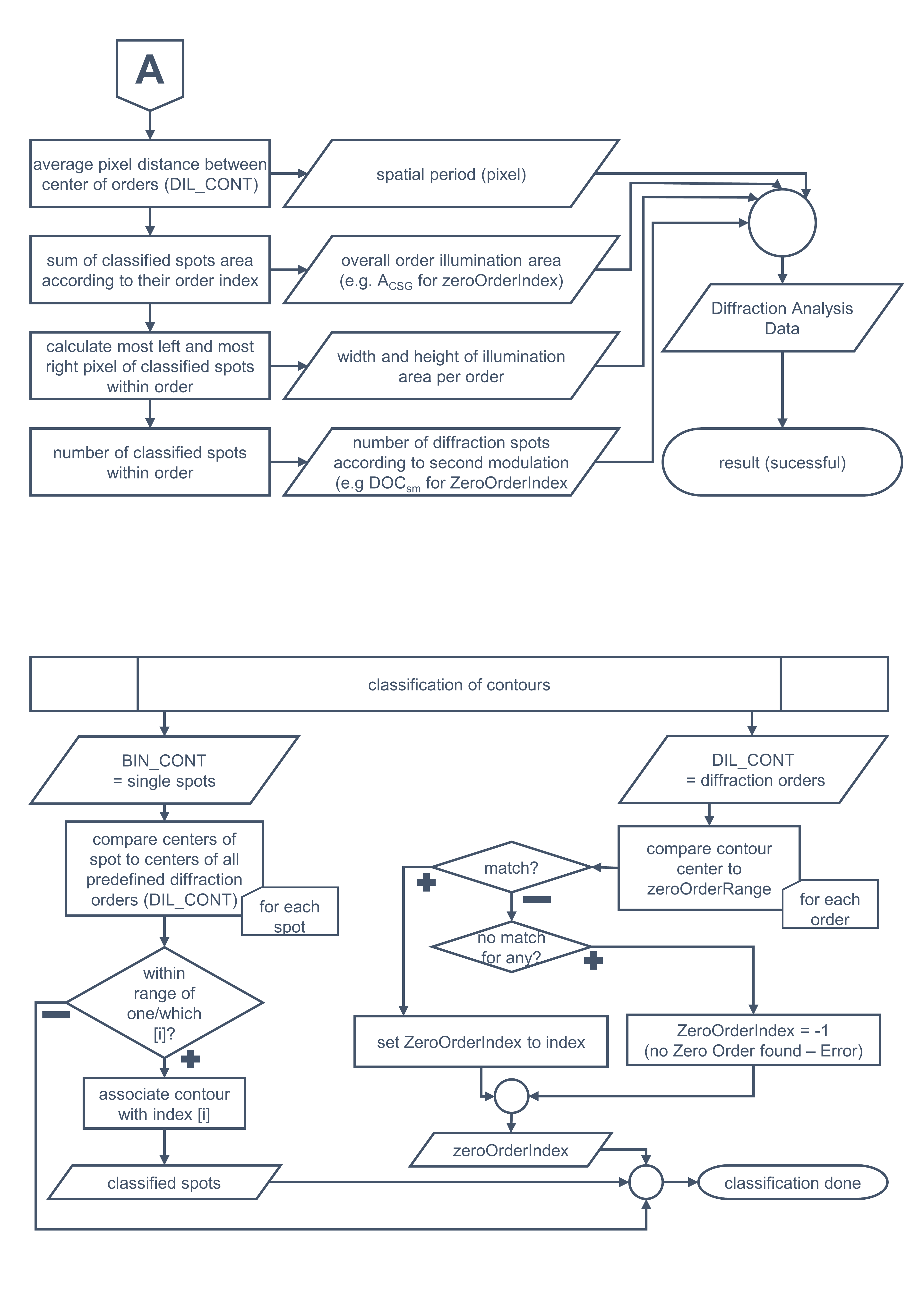

Supplement: Supplementary file 1 [file materials-13-00053-s001.zip › Figure S2 - FlowChart 2_2.TIF]

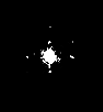

Supplement: Supplementary file 1 [file materials-13-00053-s001.zip › Figure S3 - Cat4_CSG.tif]
